# Is Revascularization the Treatment of Choice for Traumatized Necrotic Immature Teeth? A Systematic Review and Meta-Analysis

**DOI:** 10.3390/jcm12072656

**Published:** 2023-04-02

**Authors:** Mohamad Swaikat, Ignacio Faus-Matoses, Álvaro Zubizarreta-Macho, Israa Ashkar, Vicente Faus-Matoses, Carlos Bellot-Arcís, José Enrique Iranzo-Cortés, José María Montiel-Company

**Affiliations:** 1 Department of Stomatology, Faculty of Medicine and Dentistry, University of Valencia, 46010 Valencia, Spainjose.maria.montiel@uv.es (J.M.M.-C.); 2 Department of Surgery, Faculty of Medicine and Dentistry, University of Salamanca, 37008 Salamanca, Spain

**Keywords:** endodontics, revascularization, regenerative endodontics, dental trauma, necrotic immature teeth, apexification

## Abstract

Regenerative endodontic treatment (RET) has been considered a reliable procedure to treat immature necrotic teeth; however, the effect of dental trauma on the prognosis of RET is questionable. This systematic review aimed to evaluate the current level of evidence for revascularization techniques (the RET) in the management of traumatized necrotic immature permanent teeth with or without periapical radiolucent areas. Four electronic databases—PubMed, Web of Science, Scopus, and Embase—were searched until November 2022. Only randomized clinical trials, cohort studies, and case-control studies with a minimum of 10 cases and 12 months of follow-ups were included. The search identified 363 preliminary results. After discarding the duplicates and screening the titles, abstracts, and full texts, 13 articles were considered eligible. The results showed that RET techniques seemed to have high survival and success rates, 93.8% and 88.3%, respectively, in the treatment of traumatized necrotic immature permanent teeth. Root maturation with RET techniques seemed to be lower in traumatized teeth. Future studies are needed to evaluate root maturation in traumatized teeth using 3-dimensional radiographic evaluations. In addition, the lack of literature on the studies comparing RET and apexification (calcium hydroxide or an MTA) in the treatment of traumatized necrotic immature teeth highlights the necessity for high-level clinical studies comparing these treatment modalities.

## 1. Introduction

Traumatic dental injuries mainly occur in children and teenagers, and the loss of permanent teeth has lifetime consequences. Therefore, efforts should be made to preserve pulp vitality to promote continued root development and apex formation [1]. Several studies have demonstrated that pulp necrosis is the most common posttraumatic complication of all types of dental trauma [2,3]. Kaba A.D. et al. [4] found that 12% of enamel–dentin fractures produced pulp necrosis. Hecova H. et al. [3] observed that dental pulp necrosis was present in 27% of 889 permanent teeth with traumatic injuries, where the maxillary central incisors were the most frequently affected teeth; 62% of other studies showed figures close to 80% [4,5].

Pulp necrosis in immature permanent teeth leads to the discontinuation of root development, leaving the tooth with an open apex, thin dentinal walls, and short roots, making root canal debridement difficult, presenting an unfavourable crown-to-root ratio, and increasing the risk of future root fracture under occlusal forces [6,7,8]. Therefore, the optimal goal of treating these teeth is to prevent or resolve apical periodontitis, achieve continuous root development, and regenerate a dentin–pulp complex that restores the functional properties of this tissue [8].

There are three main treatment options to manage traumatized immature permanent teeth with infected root canal systems: apexification with calcium hydroxide, apexification with a mineral trioxide aggregate (MTA), and regenerative endodontic procedures [9].

The traditional approach for treating traumatized permanent teeth with open apices is apexification using calcium hydroxide (Ca(OH)_2_) dressings. However, it is a long treatment with multiple visits, which may reduce patient compliance, complicate follow-ups, and increase the vulnerability of coronal restoration [6]. In addition, Ca(OH)_2_ dressings have a significant negative effect on root strength and increase the risk of cervical fractures [10,11,12].

Recently, apexification has been performed with a mineral trioxide aggregate (MTA), also known as apexification with an MTA or an MTA apical plug [13]. An MTA is biocompatible, has the ability to set in the presence of blood, induces hard tissue formation, and has antibacterial effects due to its high pH [14,15]. In addition, MTA apexification can be performed in one or two visits, increasing patient compliance, improving the healing rate, and reducing the risk of subsequent tooth fractures [11,16]. Both apexification techniques induce the formation of an apical hard tissue barrier and promote periapical healing and the disappearance of clinical symptoms. While Ca(OH)_2_ apexification is still used by some practitioners, studies have shown that MTA apexification provides more advantages [17,18,19]. Nevertheless, it is important to highlight that apexification by either Ca(OH)_2_ or an MTA does not strengthen the root or foster further root development in terms of increasing the root length or width [11,20].

Replacing damaged root structures, as well as the pulp–dentin complex, is an optimal approach for treating traumatized immature permanent teeth with a necrotic pulp. Therefore, regenerative endodontic treatment (RET) has recently been introduced as an alternative to conventional treatment [21]. RET in immature permanent teeth has been highlighted in studies of dental trauma, with evidence that the dental pulp often remains vital despite substantial traumatic injuries such as intrusions and avulsions [22,23]. It has been radiographically proven that regeneration was possible in re-implanted teeth after intentional or traumatically related avulsion [24]. Therefore, if a favourable environment is established, the same can be accomplished in an infected tooth [25].

RET is defined as “biologically based procedures designed to replace damaged structures, including dentin and root structures, as well as cells of the pulp-dentin complex” [26]. RET is used in pain and inflammation resolution, the healing of periapical lesions, and increasing root length and thickness (maturogenesis) [27,28]. It is considered by the American Association of Endodontics [29], the European Academy of Paediatric Dentistry [30], and the European Society of Endodontics [31] as an accepted procedure to treat immature necrotic permanent teeth.

RET procedures include the following key points: (a) minimal or no instrumentation of the dentinal walls, (b) disinfection with irrigants, (c) the application of an intracanal medicament, (d) provocation of bleeding into the canal and creation of a blood clot, (e) capping with a hydraulic silicate cement, and (f) an effective coronal seal [31].

In 2011, Lovelace T.W. et al. [32] demonstrated that the evoked-bleeding act in regenerative endodontic procedures results in clinically significant delivery of mesenchymal stem cells into root canal systems, as periapical tissues in immature permanent teeth are rich in blood supply and contain stem cells that have the potential for tissue regeneration. Other studies [23,33] suggest that dental trauma injuries damage the periapical vasculature and Hertwig’s epithelial root sheath (HERS), even when the coronal dentin and enamel remain intact. These studies stated that partial removal of the HERS may compromise root development and lead to PDL and bone invasion into the pulp canal [23,33].

All previous systematic reviews conducted to evaluate the efficacy of RETs in the management of immature necrotic teeth included studies of both traumatized and non-traumatized immature necrotic teeth [34,35,36]. To date, no systematic review with meta-analysis has been conducted on the effectiveness of the RET technique in the management of traumatized immature necrotic permanent teeth. Therefore, this systematic review and meta-analysis aimed to evaluate the level of evidence of RET in the treatment of traumatized immature necrotic permanent teeth with or without periapical lesions after a minimum follow-up of 12 months and to conduct a meta-analysis on the survival and success rates of the treated teeth. The secondary objectives were to evaluate root development, apex formation, tooth vitality, and crown discoloration in the treated teeth.

## 2. Materials and Methods

### 2.1. Protocol and Registration

The present work followed the guidelines recommended by the PRISMA Statement (Preferred Reporting Items for Systematic Reviews and Meta-analysis) [37] and was performed in accordance with the current recommendations regarding endodontic systematic reviews and meta-analyses [38]. The systematic review protocol was previously registered in the Prospective Register of Systematic Reviews (PROSPERO) of the University of York with the registration number CRD42021261774.

### 2.2. Eligibility Criteria

A research question was developed in accordance with the Patient, Intervention, Comparison, and Outcome (PICO) method: “What is the current scientific evidence for pulp revascularization in the management of traumatized necrotic immature permanent anterior teeth?”, where (P): patients with traumatized necrotic immature permanent anterior teeth with or without periapical radiolucency, (I): different protocols of pulp revascularization, including blood clots, platelet-rich plasma [PRP], platelet-rich fibrin [PRF], and the platelet pellet [PP], used in the treatment of traumatized necrotic immature permanent anterior teeth, (C): traumatized necrotic immature permanent anterior teeth treated with conventional treatment, which is apexification [MTA apexification or Ca(OH)_2_ apexification], (O): the survival, clinical, and radiographic success of pulp revascularization in the treatment of traumatized necrotic immature permanent anterior teeth.

### 2.3. Outcome Measures

The primary outcomes were survival, clinical, and radiographic success. Survival was defined as a tooth that remained in the oral cavity after the follow-up. Clinical success was achieved with the absence of clinical symptoms (i.e., tenderness to percussion or palpation, swelling or fistulas, or spontaneous pain). Radiographic success was obtained when a reduction in the size of the periapical area was observed, with the absence or no increase in the size of the adverse events, such as root resorption and ankylosis, after the follow-up. Secondary outcomes were: continuation of root development, pulp vitality, and crown discoloration, where the continuation of root development had 3 aspects: increased root length, increased root width, and reduced apical diameter.

### 2.4. Study Selection Criteria

The inclusion criteria were as follows: randomized clinical trials (RCTs), retrospective and prospective cohort studies, and case-control studies. This review only included studies with a follow-up of a minimum of 12 months and studies with a minimum of 10 cases. Only articles that studied pulp revascularization (the RET) procedures or compared RET with other techniques (i.e., apexification) in traumatized necrotic immature teeth were included. Studies were not restricted by language or year of publication.

### 2.5. Databases and Search Strategy

The database search, study selection, and data extraction were performed by two independent examiners (M.S.; I.A.). In the case of any discrepancy between them, a third author was consulted (V.F.L.).

A systematic advanced electronic search was performed in PubMed, the Web of Science, Scopus, and Embase on the 21st of June 2021 and later updated on 28 November 2022, without any date or language restrictions. The search was conducted using boolean operators ‘AND’ and ‘OR’ to annex the terms and develop the search strategy. The search terms were structured as follows: (regenerative endodontic treatment OR regenerative* OR endodontic regeneration OR regenerative endodontics OR Regenerative Approach OR pulp revascularization OR revascularization* OR revitalisation* OR revitalise* OR blood clot OR platelet-rich fibrin OR platelet-rich plasma) AND (immature teeth OR Open apex OR immature dentition) AND (necrosis OR necrot* OR (non-vital AND tooth*) OR pulpless) AND (Dental trauma* OR Traumatized* OR Traumatic* OR tooth Injuries) (Supplementary Table S1). In addition, reference lists of all selected articles were screened to identify additional studies.

### 2.6. Study Selection Process

Titles and abstracts of all studies were assessed independently by two reviewers (M.S. and I.A.). When no sufficient information was provided by screening the abstract, the full article was reviewed before making the final decision. Similarly, the two examiners extracted all data regarding the relevant variables. A researcher who had not participated in the selection process (J.M.M.-C.) performed the subsequent meta-analysis.

### 2.7. Data Extraction

The variables extracted from each included article were: author and year of publication, study type, sample size, number of groups, demographic variables (gender and age), follow-up in months, loss to follow-up, type of trauma, irrigant agents used and their concentrations, root canal dressing, duration of root canal dressing in weeks, scaffold type with or without a matrix, type of coronal seal, survival rate, success rate, failure, changes in root length and width, changes in apical diameter, adverse events or effects, crown discoloration, and tooth.

### 2.8. Methodological Quality Assessment

Two researchers assessed the risk of bias in all selected studies (M.S. and J.M.M.-C.) using a Cochrane risk-of-bias tool, RoB 2.0 [39], to evaluate randomized clinical trials and the ONS (Newcastle–Ottawa scale) [40] to evaluate non-randomized studies, including case-control and cohort studies.

The NOS includes 8 items with a potential score of 9. Three main domains are considered: patient selection, comparability of the study groups, and results or outcome. Articles were classified as being of ‘high’, ‘moderate’, or ‘low quality’, where high-quality articles scored more than 6 points.

The Cochrane RoB 2.0 methodology assessment [39] consists of five domains that evaluate: the randomization process, deviations from intended interventions, missing outcome data, outcome measurement, and selection of reported outcomes. Producing three levels of bias: ‘low risk of bias’, ‘some concerns’, or ‘high risk of bias’.

### 2.9. Quantitative Synthesis—Meta-Analysis

All studies included in the meta-analysis were combined using a random-effects model. The estimated effect size was the event rate, odd ratios, means, and different means. The 95% confidence intervals were calculated for all estimated variables.

Heterogeneity among the combined studies was assessed using a Q test (*p*-value < 0.05) and quantified with the I^2^, considering slight heterogeneity if it was 25–50%, moderate if 50–75%, and high heterogeneity if > 75%. Statistical significance was tested using a Z test (*p*-value < 0.05). The meta-analysis has been represented with a forest plot, and the publication bias was assessed using an Egger’s test that indicates the existence of possible publication bias when the *p*-value is less than 0.05 (indicating significant asymmetry).

## 3. Results

### 3.1. Study Selection

**Figure 1:**
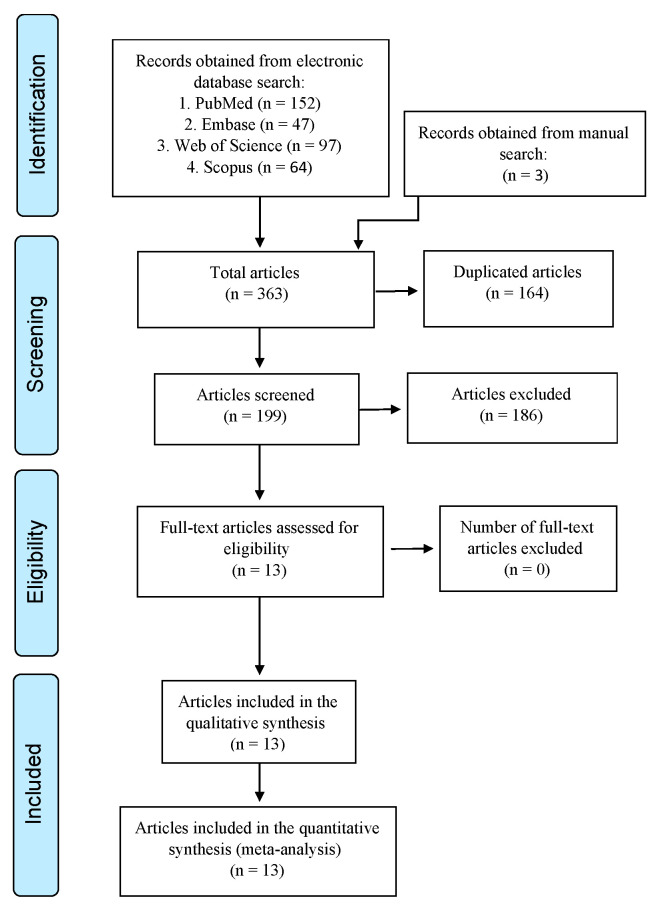
Preferred Reporting Items for Systematic Reviews and Meta-Analyses (PRISMA) flow diagram.

The search identified a total of three hundred and sixty-three preliminary results, where one hundred and fifty-two were found in PubMed, forty-seven in Embase, ninety-seven in the Web of Science, sixty-four in Scopus, and three from a manual search of reference lists. The duplicates were manually removed using Mendeley reference management software, and a total of 199 studies remained. After the title and abstract screening, 186 articles were excluded. A total of 13 studies were eligible for full-text reading, and all were selected for qualitative and quantitative synthesis (Figure 1).

### 3.2. Characteristics of the Studies

The sample size of the selected studies ranged from 15 [41] to 118 cases [42]. The patients’ gender was mentioned in most studies, except in three [42,43,44]. The minimum follow-up time was 12 months [42,43,45,46,47], and the maximum follow-up time was “43.42” months [41]. As for dropouts, five studies had no dropouts [44,45,48,49,50], and the other six studies [41,42,43,51,52,53] had dropouts that ranged from 1 [51] to 16 [47]. The results are presented in Table 1 and Supplementary Table S2.

### 3.3. Assessment of Risk of Bias

Six RCTs were identified, and the Cochrane RoB 2 tool was used for quality assessment. Only one study [46] was considered as a ‘low risk of bias’, four studies were considered as ‘Some concerns’ [42,43,44,53], and one study had a ‘high risk of bias’ [48] (Table 2). The quality was mainly affected by non-compliance with the items related to the blinding of patients or evaluators. As for the rest of the studies, NOS was used for the quality assessment, and one case-control study was identified with ‘moderate’ methodological quality [a score of 5] [49], four cohort studies with ‘moderate’ methodological quality [a score of 5–6] [41,45,50,51], and two cohort studies with ‘high’ methodological quality [a score of 7] [47,52] (Table 3).

The articles were classified as being of ‘high’, ‘moderate’, or ‘low’ quality, where high-quality articles scored more than six points.

### 3.4. Quantitative Analysis Results

#### 3.4.1. Survival and Success Rate

**Figure 2:**
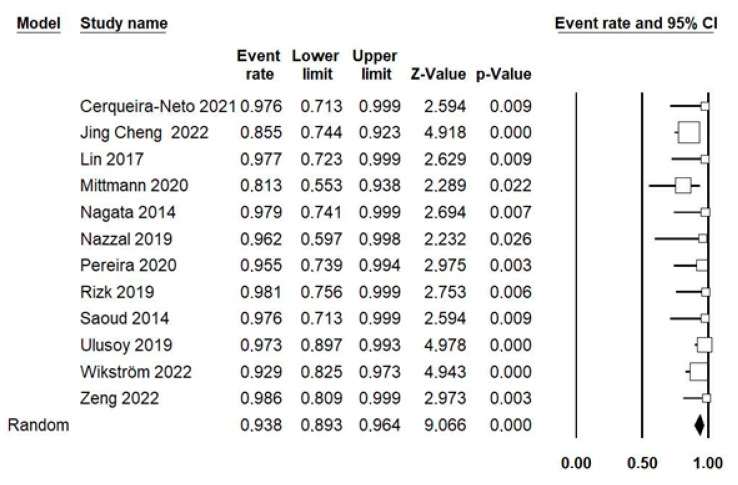
Forest plot of the estimated survival rate of traumatized teeth treated with pulpal revascularization among the selected studies. Using a random-effects model where 12 studies, including 384 cases, were combined to estimate the survival rate of RET [42,44,45,46,47,48,49,50,51,52,53,54].

**Figure 3:**
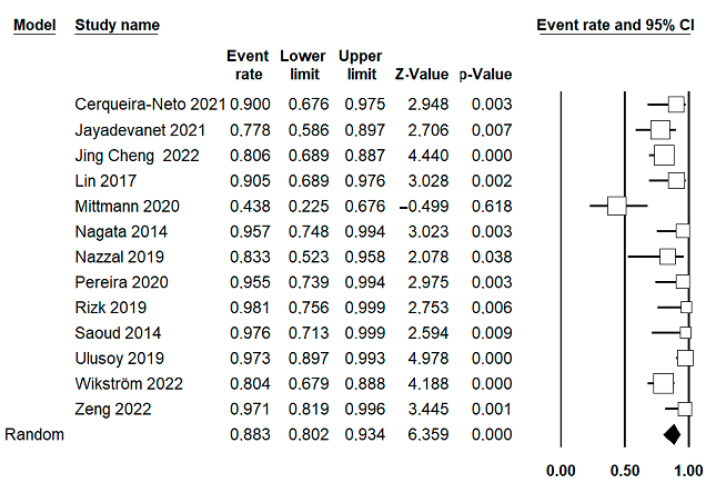
Forest plot of the estimated success rate of traumatized teeth treated with pulpal revascularization among the selected studies. Using a random-effects model where 13 studies, including 411 cases, were combined to estimate the success rate of RET [42,43,44,45,46,47,48,49,50,51,52,53,54].

Using the random-effects model, 12 studies, including 384 cases, were combined to estimate the survival rate, and 13 studies, including 411 cases, were combined for the success rate. The estimated survival rate for traumatized teeth treated with RET was 93.8% (95% CI; 89.3–96.4%), with a slight heterogeneity between the combined studies (*p* = 0.183; I^2^ = 26.6%; Q-value = 14.9) (Figure 2). The estimated success rate was 88.3% (95% CI; 80.2–93.4%), with a moderate heterogeneity between the combined studies (*p* < 0.001; I^2^ = 66.7%; Q-value = 36) (Figure 3).

#### 3.4.2. Root Development, Apex Formation, Tooth Vitality, and Crown Discoloration

The results of the meta-analysis, with the number of combined studies, samples, and heterogeneity for signs of apical closure (partial or complete), the decrease in apical diameter, increase in root length and width, regain of vitality, and crown discoloration, are listed in Table 4.

#### 3.4.3. Apexification and RET Comparison

**Figure 4:**
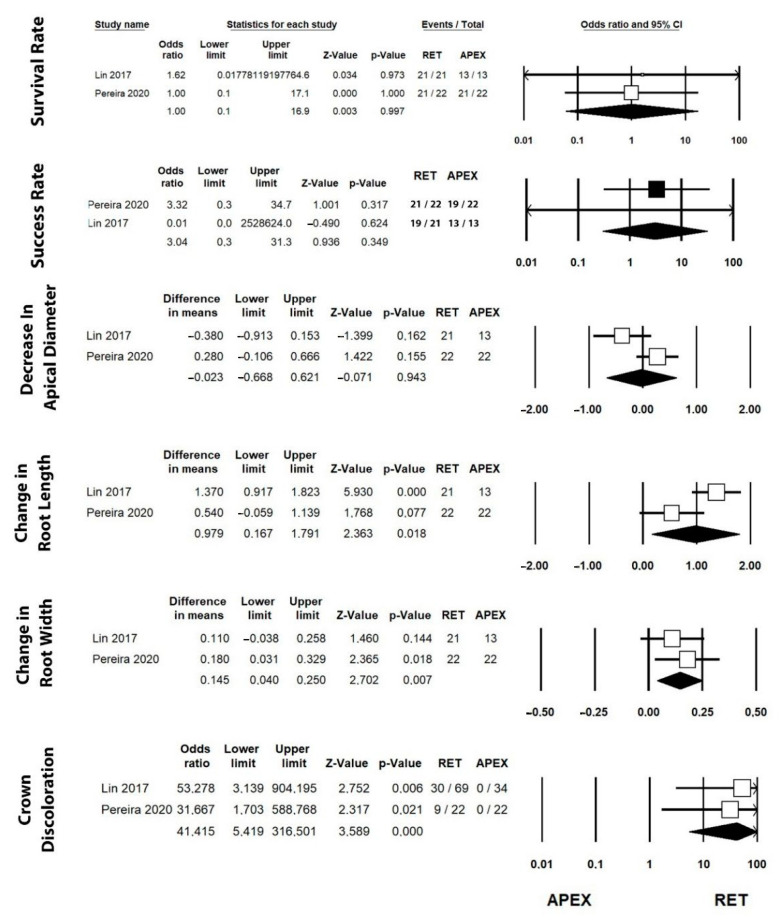
Forest plot of estimated odds ratio and difference in means for the studied variables comparing RET and apexification techniques. Results of two studies that compared the apexification and RET techniques [42,49].

Only two studies [42,49] compared both the apexification and RET techniques. The odds ratios and differences in means for survival and success rate, increase in root length and width, apex formation, and crown discoloration are presented in Figure 4.

### 3.5. Publication Bias

The results of the Egger test used to assess the publication bias are presented in Supplementary Table S3.

## 4. Discussion

Previous studies reported that the success rate of RET is lower in traumatized immature teeth [51,54,55] in comparison to RET in non-traumatic dental injuries, such as caries or developmental anomalies [20,27,42]; however, a recent systematic review and meta-analysis demonstrated that there is no relationship between the aetiology of pulp necrosis in immature teeth and the success rate after RET [35], which is aligned with the results of a study conducted by Linsuwanont et al. [56]. Among the included studies in our review, Mittmann et al. [51] reported the lowest survival and success rates of RETs (81.3% and 44%, respectively); this could be associated with the severity of the trauma as 75% of the cases were serious avulsion, 19% were luxation, and 6% were intrusion cases. Similarly, Jing Cheng et al. [50] reported that failure of RET occurred in avulsed teeth more than in other types of traumatic teeth. In addition, Wikström et al. [52] stated that the failed 11 cases were characterized by severe trauma. Hence, severe trauma might influence the outcome of RET when compared with mild-to-moderate trauma as, according to some studies and depending on the type of dental trauma, irreversible damage to the HERS may be present in traumatized immature teeth affected by different degrees of traumatic injuries [22,23]. The quantitative analysis in our review estimated that the survival and success rates of necrotic traumatized immature permanent teeth treated with RET and followed up after 12–48 months were 93.8% and 88.3%, respectively. Our results are slightly lower than the findings of Torabinejad et al. [57], who previously reported a survival rate of 97.8% and a success rate of 91.3%; the slight difference in the results may be due to the fact that the aforementioned study included all teeth treated with RET regardless of the aetiology of the pulp necrosis.

In this systematic review, to quantify the continuation of root development, most studies measured, over time, the radiographic changes in the root after RET. Specifically, the prognosis of root maturation was evaluated according to the following parameters: the number or percentage of cases with signs of root maturation, the total mean percentage of root maturation after the follow-up, and the total mean of root development in millimetres. Kim S.G. et al. [58] suggested that traumatic injuries can negatively affect the success of root maturation after RET due to the interaction between the HERS and mesenchymal stem cells in the dental follicle. Similarly, Zeng et al. [47] demonstrated that the aetiology of pulp necrosis (dens evaginatus or trauma) has a significant effect on root development after RETs and that traumatized immature teeth have a lower root development success rate. In a recent review [34], immature necrotic teeth treated with RET, regardless of the aetiology of the pulp necrosis, reported an 80% success rate of dentin thickening and lengthening; however, in our review, all the included studies reported a lower success rate of root development, and the results of root maturation among the studies varied, as six studies [42,46,48,49,51,52] exhibited more increase in the root length over the root width, and seven studies [41,43,44,45,47,50,53] reported more increase in the root width over the root length. Nevertheless, only six of the included studies [41,42,48,49,52] quantified the continuation of root development in millimetres, and our quantitative analysis of these studies estimated a 1.26 mm mean increase in root length and a 0.52 mm mean increase in root width.

The variation in the results could be attributed to the heterogeneity among the studies, which could be caused by the variability in the participants, studied interventions and outcomes, outcome measurement tools, and risk of bias [59]. Moreover, 11 out of 13 included studies [41,43,44,45,48,49,50,51,52,53] used 2-dimensional radiographs (periapical radiographs) to evaluate the outcome of root development, and only two studies used a cone-beam computed tomography (CBCT) scan [42,47]. Taking into consideration the limitations of 2-dimensional radiographs, since the root development occurs in a 3-dimensional (3D) pattern, 3D imaging is the most accurate method for the assessment of root development and patterns of hard tissue formation [60]. Although Flake N.M. et al. [61] recently introduced a standardized method to measure radiographic root changes in 2-dimensional radiographs after RET, it has been shown that dental trauma can be better assessed in three dimensions [62]. CBCT has been demonstrated to be a more powerful diagnostic tool for evaluating the anatomical characteristics in a three-dimensional pattern to know, in advance, the measurements of the last apical millimetre of the roots. [63].

Previous studies [21,32] demonstrated that stem cells from the apical papilla (SCAP) are essential in regenerative endodontic procedures, and their conservation should be a priority for clinicians. Martin D.E. et al. [64] reported that different concentrations of NaOCl can influence the survival rate of stem cells and recommended a concentration of NaOCl between 1.5 and 3% during RET. The included studies in the present systematic review used different NaOCl concentrations: 0.5% [41,52], 1% [51,53], 1.5% [42,43,47,50], 2% [46,49], 2.5% [45], 5% [44], and 6% [48]; therefore, lower or higher concentrations of NaOCl may have negatively influenced the outcome of the RET. Previous studies recommended the use of ethylenediaminetetraacetic acid (EDTA) in the second follow-up appointment of RET [29,31,64,65], as it reduces the undesirable effect of NaOCl on stem cell viability and releases growth factors. Although, another study by Farhad Mollashahi et al. [66] demonstrated that EDTA had higher cytotoxicity than NaOCl on the survival of stem cells of the human apical papilla and that CHX had less cytotoxicity compared with NaOCl or EDTA. In addition, a recent systematic review reported that there were no failed cases when EDTA was not used as an irrigation solution during RET procedures [35]. In the present review, two of the included studies [41,45] did not irrigate with EDTA and still had similar results to the other included studies. Further investigations regarding the use of EDTA in RET are required to better understand its impact on such treatments.

Adequate disinfection of the root canal system is an essential step in RET [67]. Triple antibiotic paste (TAP) has been highlighted as an effective antimicrobial intracanal medicament in RET procedures, but it has been shown to be cytotoxic to the stem cells of the apical papilla at clinical concentrations [65,68]. Berkhoff et al. [67] reported that a TAP presented high diffusion and retention within the dentin and that 80% of this intracanal agent cannot be removed in the second appointment of RET, regardless of the irrigation agent used. Therefore, current guidelines recommend the use of low-concentration antibiotic mixtures or calcium hydroxide [29,31], as Nagata et al. [44] made a comparison between TAP and Ca(OH)_2_ and reported that there were no clinical or radiographic differences between the two aforementioned protocols. In the present review, all of the studies, except for two [48,52], used TAP as the intracanal medicament for RET for a dressing time from 7 days [51] to 4 weeks [43,47,53] and had a similar outcome to the rest of the studies, although the number of studies is inadequate to make a clear conclusion regarding this parameter.

Tooth discoloration after RET was reported in seven of the included studies [41,42,44,48,50,51,52], and our analysis estimated that this event was presented in 37.9% of traumatized immature necrotic teeth treated with RET. Crown discoloration may be attributed to the use of TAP as the intracanal medicament [69,70,71], as tetracycline-based antibiotics (minocycline or doxycycline) can result in higher crown discoloration [71,72], which is why some studies [70,71] suggest the use of amoxicillin-based antibiotics or double antibiotic paste (DAP), as it induces less coronal discoloration. On the other hand, the bismuth oxide content in an MTA is also associated with coronal discoloration [72,73]. Therefore, some studies [74,75] suggested the use of dentin bonding agents and a flowable composite in the coronal pulpal chamber; nevertheless, the efficacy of this technique has not been confirmed yet.

The results of the present review estimated that 16.2% of the traumatized necrotic immature teeth would respond to sensibility after the RET. All of the studies in this review used cold and electric pulp sensibility tests, while only two studies used thermal tests [45,46]. Ulusoy et al. [53] reported that a “blood clot” study group had a significantly slower initial response to sensitivity tests and suggested that the higher platelet level of the biological scaffolds may have stimulated the regeneration process of the sensory fibres; these results were in agreement with two previous studies [73,76]. However, Rizk et al. [46] and Jayadevan et al. [43] used PRP and PRF scaffolds and reported no response to pulp sensibility tests in all of the cases, which agrees with Torabinejad et al. [77]. The negative responses might have arisen for different reasons: First, in the children population, it was difficult to obtain accurate pulp testing scores. Second, the thick three-layered coronal seal over the “blood clot” scaffold. Third, dentin sensitivity in the natural pulp–dentin complex is related to the hydrodynamic activity of dentin tubules in association with A-β sensory fibres, whereas newly regenerated, mineralized root canal tissues do not appear to have well-organized dentin tubules and, thus, may not have exhibited the same sensitivity as natural tissue [78]. Therefore, the results of the sensibility tests should be interpreted with caution.

This review reports the necessity to carry out more comparative studies between the apexification technique (with Ca(OH)_2_ or an MTA) and RET procedures for the treatment of necrotic traumatized immature permanent teeth. To date, there are only two studies [42,49] that compared these two techniques, and one of them is not randomized. The lack of randomized clinical trials comparing these two treatment modalities makes the comparisons less conclusive.

Within the limitations of our study, most of the included randomized studies have a risk of bias of “some concerns”, and seven out of thirteen studies were non-randomized with moderate or low quality and an unclear risk of bias. In addition, there is no standardized protocol for RET used in all of the included studies, which causes heterogeneity among the studies. Moreover, there were variations in the assessment criteria and interpretation of the results among the studies, and most of the studies have a small sample size and lack long-term follow-ups, which are important to address an accurate assessment of the RET outcome. Therefore, the result of this review must be interpreted with caution. Additional long-term, high-quality clinical studies with larger sample sizes, standardized protocols, and accurate radiographic evaluations, such as by CBCT scans, are needed to evaluate the success of RET techniques in the management of necrotic traumatized immature teeth and root maturation. The lack of literature on studies comparing RET and apexification (Ca(OH)_2_ or an MTA) in the treatment of necrotic traumatized immature teeth addresses the necessity for high-level clinical studies comparing these two treatment modalities.

## 5. Conclusions

Based on the present review and meta-analysis, RET techniques seem to have high survival and success rates in the treatment of traumatized necrotic immature permanent teeth, and they appear to be similar to those immature teeth affected by caries or dental anomalies. However, severe trauma may have a negative effect on the results of RET treatment, and further future studies are needed to support this hypothesis.

Root maturation (the lengthening and thickening of the dental walls) with RET techniques seems to be less in traumatized teeth. Nevertheless, future studies are needed to evaluate root maturation in traumatized teeth using 3-dimensional radiographic evaluations.

Coronal discoloration is an adverse event in most traumatized teeth treated with RET, so internal bleaching might be indicated at the end of the treatment, and future clinical studies are needed to overcome this issue.

The recuperation of sensitivity after RET is not clear due to difficulties in counting pulp vitality tests as an accurate method for evaluation.

## Figures and Tables

**Table 1:** Methodological characteristics of the included studies.

Study	Design	Sample (n)	Gender (M/F), Age (y-Old) (Range) *M* (Mean)	Follow-Up Time (Months)	Dropouts	Type of Trauma [n], (%)	Irrigant Used		Root Canal Dressing, Duration (Weeks or Days)	Scaffold Type with or without Matrix	Type of Coronal Seal
							1st Visit	2nd Visit			
**Cerqueira-Neto, 2021** [48]	CA.CO	n = 20; 2 groups: - n = 11 - n = 9	10 M, 7 F (7–15) *M* = 9.4	24	0	FRA [1] LUX [5] FRUX [13] REIMP [1]	G.: 6% IND NaOCl, sterile 5% sodium thiosulfate, saline, 2% CHX, 17% EDTA	G. IND: saline, 17% EDTA	G. IND Ca (OH)₂ and 2% CHX gel (3 weeks)	BC & CB	Mixture of Ca (OH)₂, 2% CHX, and zinc oxide
							G.SV: 6% NaOCl, sterile 5% sodium thiosulfate, saline, 2% CHX, 17% EDTA	----	----		
**Jayadevan, 2021** [43]	RCT	n = 30; 2 groups: - A-PRF n = 15 - PRF n = 15	N.S. (8–27)	12	3	N. S.	1.5% NaOCl, saline, 17% EDTA	Saline, 17% EDTA	TAP (Cipro, Metro, and Cef) (4 weeks)	A-PRF or PRF	Biodentine
**Jing Cheng, 2022** [50]	CO. RETRO	n = 62	34 M, 28 F *M* = 8.6 ± 1.4	(6–69) *M* = 22.3	0	FRA [39] (62.9%) LUX [6] (9.7%) FRUX [14] (22.6%) AV [3] (4.8%)	0.5–1.5% NaOCl, saline	0.5–1.5% NaOCl, saline, 17% EDTA	TAP (Cipro, Metro, and Mino) or Ca (OH)₂ (2 weeks)	BC & CGF	MTA
**Lin, 2017** [42]	RCT	n = 118; 4 groups: -APEX T n = 15, -APEX DA n = 23 -RET: T n = 27 -RET DA n = 53	N.S. (6–18) *M* = 10.5 ± 1.8	12	15	N. S.	G.RET: 1.5% NaOCl, saline, 17% EDTA	Saline, 17% EDTA	G.RET TAP (Cipro, Metro, and Clinda)	BC & CB	MTA
							G. APEX: 1.5% NaOCl, saline, 17% EDTA	----------	G. APEX: Ca (OH)₂		
**Mittmann, 2020** [51]	CO. RETRO	n = 17	5 M, 6 F (6–11) *M* = 10	(11–54) *M* = 22	1	AV (75%) LUX (19%) INT (6%)	1% NaOCl, 17% EDTA	17% EDTA	TAP (Tetra and Triam) (7–10 days)	BC & CB	MTA
**Nagata, 2014** [44]	RCT	n = 23; 2 groups: - TAP n= 12 - CHP n = 11	N.S. (7–17)	(9–19) *M* = 15	0	L.LUX (47.8%) EX.LUX (39.1%) INT.LUX (4.3%) AV (8.7%)	5% NaOCl, 2% CHX, saline	Saline, 17% EDTA	G.TAP: mixture of Cipro 250 mg, Metro 400 mg, and Mino 50 mg (3 weeks)	BC & CB	MTA
									G.CHP: mixture of Ca (OH)₂ and 2% CHX gel (3 weeks)		
**Nazzal 2020** [41]	CO.PRO	n = 15	3 M, 12 F (7–10) *M* = 8.3	(27–59) *M* = 43.4	3	AV (13%) INT (7%) E/D (80%)	0.5% NaOCl	Saline	TAP (Metro (100 mg) and Cipro (100 mg)) (2 weeks)	BC	MTA
**Pereira, 2021** [49]	CA.CO	n = 44; 2 groups: - APEX n = 22 - RET n = 22	19 M, 18 F (6–18) *M* = 9.4 ± 2.2	APEX (12–24) *M* = 17.7	0	APEX: FRA (4.5%) LUX (31.8%) FRUX (54.5%) REIMP (9.1%)	APEX: 2% CHX, 17% EDTA, saline	-----	G. APEX: Ca (OH)₂, 2% chlorhexidine gel, and zinc oxide	-----	-----
				RET (12–30) *M* = 20.5		RET: LUX (22.7%) FRUX (63.6%) REIMP (13.6%)	RET: 6% NaOCl, 2% CHX, saline, EDTA 17%	RET: saline, 17% EDTA	G.RET: 6 cases with TAP (mixture of Cipro 250 mg, Metro 400 mg, and Mino 50 mg); 16 cases with Ca (OH)₂ and 2% CHX gel (3 weeks)	BC & CB	MTA
**Rizk et al., 2019** [46]	RCT	n = 26; 2 groups: - PRP n = 13 - PRF n = 13	13 M, 12 F (8–14) *M* = 9 ± 1	12	1	PRP: E/D/P (53.8%) E/D (46.2%)	2% NaOCl, 17% EDTA	Saline, 17% EDTA	TAP (Cipro, Metro, and Mino) (3 weeks)	PRP, PRF & CB	MTA
						PRF: E/D/P (91.7%) E/D (8.3%)					
**Saoud et al., 2014** [45]	CO. RETRO	n = 20	14 M, 6 F *M* = 11.3 ± 1.9	12	0	E/D (10%) E/D/P (80%) No loss structure (10%)	2.5% NaOCl, saline	Saline	TAP (Cipro 200 mg, Metro 500 mg, and Mino 100 mg) (2 weeks)	BC	MTA
**Wikström, 2022** [52]	CO.PRO	n = 63	36 M, 20 F (6–22) *M* = 10.4 ± 2.8	(24–52)	7	CON [5] (8.9%) S.LUX [6] (10.7%) L.LUX [24] (42.9%) INT [4] (7.1%) AV [10] (17.9%) E/D [39] (69.6%) Combined [35] (62.5%) Unknown [3] (5.4%)	0.5% NaOCl, saline, 17% EDTA	0.5% NaOCl, saline, 17% EDTA	Ca (OH)₂ or 2% or CHX digluconate	BC & CB	MTA
**Ulusoy, 2019** [53]	RCT	n = 88; 4 groups, - PRP n = 18 - PRF n = 17 - PP n = 17 - BC n = 21	44 M, 33 F (8–11) *M* = 9 ± 1	(10–49) *M* = 28.3 ± 1.2	15	N. S.	1.25% NaOCl, saline	2% NaOCl, saline, EDTA 17%	TAP (equal amounts (20 mg) of Clinda, Cipro, and Metro) (4 weeks)	PRP, PRF, PP, & BC	MTA
**Zeng, 2022** [47]	CO. RETRO	n = 132; 2 groups T n = 34 DA n = 82	52 M, 58 F Trauma *M* = 8.9 D. Evaginatus M = 10.9	12	16	Incisors with trauma [34]	1.5% NaOCl, 17% EDTA	1.5% NaOCl, 17% EDTA	TAP (Clinda, Cipro, and Metro) (4 weeks)	BC & CB	MTA

*M*: mean, CO. RETRO: retrospective cohort study, CO. PRO: prospective cohort study, CA. CO: case-control study, ECAs: randomized controlled trial, G. APEX: apexification group, G.RET: revascularization group, M: male, F: female, G. IND: interappointment dressing group, G.SV: single-visit group, AV: avulsion, LUX: luxation, INT: intrusion, E/D: enamel–dentin fracture, E/D/P: enamel–dentin–pulp fracture, REIMP: re-implantation, FRA: fracture, FRUX: fracture and luxation, CON: concussion, S.LUX: subluxation, TAP: triple antibiotic paste, Metro: metronidazole, Cipro: ciprofloxacin, Clinda: clindamycin, Mino: minocycline, Tetra: tetracycline, Triam: triamcinolone, Cef: cefaclor, Ca(OH)₂: calcium hydroxide, CHX: chlorhexidine gel, G.CHP: calcium hydroxide and chlorhexidine gel group, L.LUX: lateral luxation, EX.LUX: extrusive luxation, INT.LUX: intrusive luxation, DAs: dental anomalies, T: dental trauma, PRP: platelet-rich plasma, PRF: platelet-rich fibrin, PP: platelet pellet, A-PRF: advanced platelet-rich fibrin, BC: induced blood clot, CB: collagen barrier, CGF: concentrated growth factor, N.S.: not indicated.

**Table 2:** Cochrane RoB 2 tool for randomized clinical trials.

	Randomization Process	Deviations from the Intended Interventions	Missing Outcome Data	Measurement of the Outcome	Selection of the Reported Result	Overall Risk of Bias
Cerqueira-Neto, 2021 [48]	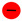	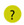	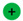	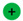	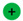	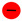
Jayadevan, 2021 [43]	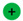	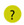	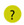	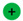	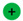	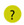
Lin, 2017 [42]	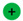	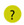	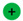	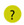	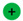	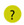
Nagata, 2014 [44]	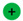	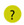	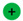	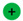	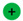	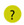
Rizk, 2020 [46]	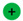	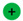	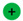	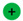	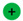	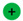
Ulusoy, 2019 [53]	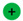	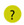	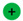	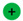	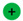	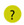

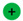
: Low Risk, 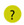: Some concerns, 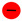: High risk.

**Table 3:** Newcastle–Ottawa scale for cohort and case-control studies.

Cohort Studies	Selection (★★★★)				Comparability (★★★)	Outcome (★★★)			Score (Out of 9)
	Item 1	Item 2	Item 3	Item 4	Item 5	Item 6	Item 7	Item 8	
Jing Cheng, 2022 [50]	★		★	★		★	★	★	6★
Mittman, 2020 [51]	★		★	★		★	★	★	6★
Nazzal, 2019 [41]	★		★	★		★	★		5★
Saoud, 2014 [45]	★		★	★		★	★	★	6★
Wikström, 2022 [52]	★		★	★	★	★	★	★	7★
Zeng, 2022 [47]	★		★	★	★	★	★	★	7★
Case-Control studies	Selection (★★★★)				Comparability (★★★)	Outcome (★★★)			Score (Out Of 9)
	Item 1	Item 2	Item 3	Item 4	Item 5	Item 6	Item 7	Item 8	
Pereira 2021 [49]	★	★		★		★	★		5★

★: tool is available or described. In cohort studies, Item 1: representativeness of the exposed cohort; Item 2: selection of the non-exposed cohort; Item 3; Item 4: demonstration that outcome of interest was not present at the start of the study; Item 5: comparability of cohorts on the basis of the design or analysis; Item 6: assessment of outcome; Item 7: was the follow-up long enough for outcomes to occur?; Item 8: adequacy of follow-up of cohorts. In case-control studies, Item 1: is the case definition adequate?; Item 2: representativeness of the cases; Item 3: selection of controls; Item 4: definition of controls; Item 5: comparability of cases and controls on the basis of the design or analysis; Item 6: ascertainment of exposure; Item 7: same method for ascertainment for cases and controls; Item 8: non-response rate.

**Table 4:** Results of meta-analysis for all studied variables.

Studied Variable	Number of Combined Studies	N	Mean or Event	Heterogeneity		
				*p*-Value	I^2^	Q-Value
Survival Rate	12	384	93.8% (95% CI, 89.3–96.4%)	0.183	26.6	14.9
Success Rate	13	411	88.3% (95% CI, 80.2–93.4%)	<0.001	66.7	36
Signs of Apical Closure Event	6	216	75.9% (95% CI, 61.8–86.1%)	0.006	69.2	16.2
Decrease in Apical Diameter	5	100	1.51 mm (95% CI, 0.95–2.07 mm)	<0.001	85.1	26.9
Increase in Root Length	6	145	1.26 mm (95% CI, 1.07–1.46 mm)	0.181	33.9	7.57
Increase in Root Width	6	145	0.52 mm (95% CI, 0.26–0.78 mm)	<0.001	92.3	64.9
Regain of Vitality	10	279	16.2% (95% CI, 4.5–44.1%)	<0.001	90.2	92.5
Crown Discoloration	7	185	37.9% (95% CI, 21.9–57%)	<0.001	75.5	24.5

N: cumulative sample size; I^2^: % of total variability due to heterogeneity. Heterogeneity among the combined studies was assessed using a Q test (*p*-value < 0.05) and quantified with the I^2^, where heterogeneity is considered slight if I^2^: 25–50%, moderate if I^2^: 50–75%, and high if I^2^ > 75%. Statistical significance was tested using a Z test (*p*-value < 0.05).

## Data Availability

The data presented in this study are available in the article and Supplementary Material.

## Supplementary Materials

The following supporting information can be downloaded at: https://www.mdpi.com/article/10.3390/jcm12072656/s1, Table S1: search strategies for each database; Table S2: results of RET in each included study; Table S3: results of the Egger test of each RET meta-analysis performed.
