# Supplementary material for: Is Revascularization the Treatment of Choice for Traumatized Necrotic Immature Teeth? A Systematic Review and Meta-Analysis
Source: J Clin Med. 2023 Apr 2;12(7):2656. doi: 10.3390/jcm12072656 (PMC10095182; doi:10.3390/jcm12072656)
Supplement: Supplementary file 1 [file jcm-12-02656-s001.zip › jcm-2305289 -supplementary.pdf]

Table S1. Search strategies for each database.

Database	Search strategy	Findings
**Pubmed**	(regenerative endodontic treatment OR regenerative* OR endodontic regeneration OR regenerative endodontics OR Regenerative Approach OR pulp revascularization OR revascularization* OR revitalisation* OR revitalise* OR blood clot OR platelet-rich fibrin OR platelet-rich plasma) AND (immature teeth OR Open apex OR immature dentition) AND (necrosis OR necrot* OR (non-vital AND tooth*) OR pulpless) AND (Dental trauma* OR Traumatized* OR Traumatic* OR tooth Injuries).	152
**Scopus**	(TITLE-ABS-KEY ("regenerative endodontic treatment" OR regenerative* OR "endodontic regeneration" OR "regenerative endodontics" OR "regenerative approach" OR "pulp revascularization" OR revascularization* OR revitalisation* OR revitalise* OR "blood clot" OR "platelet rich fibrin" OR "platelet-rich plasma") AND TITLE-ABS-KEY ("immature teeth" OR "open apex" OR "immature dentition") AND TITLE-ABS-KEY (necrosis* OR necrot* OR "non-vital tooth" OR pulpless) AND TITLE-ABS-KEY ("dental trauma*" OR traumatized* OR traumatic* OR "tooth injuries")).	64
**Embase**	('regenerative endodontic treatment' OR regenerative* OR 'endodontic regeneration' OR 'regenerative endodontics' OR 'regenerative approach' OR 'pulp revascularization' OR revascularization* OR revitalisation* OR revitalise* OR 'blood clot' OR 'platelet-rich fibrin' OR 'platelet-rich plasma') AND ('immature teeth' OR 'open apex' OR 'immature dentition') AND (necrosis OR necrot* OR 'non-vital tooth*' OR pulpless*) AND ('dental trauma*' OR traumatized* OR traumatic* OR 'tooth injury')	47
**Web of science**	((((((((((TS=(regenerative endodontic treatment)) OR TS=(regenerative*)) OR TS=(endodontic regeneration)) OR TS=(regenerative endodontics)) OR TS=(Regenerative Approach)) OR TS=(pulp revascularization)) OR TS=(revascularization*)) OR TS=(revitalisation*)) OR TS=(revitalise*)) OR TS=(blood clot)) OR TS=(platelet-rich fibrin)) OR TS=(platelet-rich plasma) AND ((TS=(immature teeth)) OR TS=(Open apex)) OR TS=(immature dentition) AND (((TS=(necrosis)) OR TS=(necrot*)) OR TS=(non-vital AND tooth*)) OR TS=(pulpless) AND (((TS=(Dental trauma*)) OR TS=(Traumatized*)) OR TS=(Traumatic*)) OR TS=(tooth Injuries).	97

Table S2. Results of RET in each included study.

Study	Survival Rate	Success Rate	Failure	Increase In Root Length (Mean)	Increase In dentinal wall thickness	Decrease In Apical Diameter	Crown Discoloration (%)	Regain of Vitality (%)	Adverse Events
Cerqueira-Neto et al., 2021 [48]	20/20 (100%)	18/20 (90%)	2 cases (10%)	G. IND: 0.97 mm ± 0.41 (7.89 6% ± 4.20)<br>G. SV: 1.59 mm ± 0.91 (11.27% ± 7.34)<br>Total: 1.28 mm ± 0.66 (9.58% ± 5.77)	G. IND: 0.58 mm ± 0.22 (20.49% ± 8.47)<br>G. SV: 0.62 mm ± 0.38 (18.97% ± 12.70)<br>Total: 0.60 mm ± 0.30 (19.73% ± 10.59)	G. IND: 2.13mm ± 1.09(100 % ± 0)<br>G. SV: 2.34 mm ± 0.82 (100% ± 0)<br>Total: 2.24 mm ± 0.96 (100% ± 0)	0/20	4/20	---------
Jayadevan et al., 2021 [43]	---------	21/27 (77.7%)	6 cases (22.3%)	A-PRF: 1.6%<br>PRF: 2.17%	A-PRF: 14.29%<br>PRF: 3.95%	---------	N. S	0/21	---------
Jing Cheng et al., 2022 [50]	53/62 (85.5%)	50/62 (80.6%)	12 cases (19.4%)	10.2% ± 13.7	22.6% ± 24.7	69.3% ± 35.3	15/50	7/50	REAB 4/62 (6.5%)
Lin et al, 2017 [42]	21/21 (100%)	19/21 (90%)	--------	G.RET: mean of 0.68 ± 1.29 (mm)	G.RET: mean of 0.19 ± 0.18 (mm)	G.RET: mean of 1.30 mm ± 0.86	-----------	N. S	REAB 2/21 (10%)
				G. APEX: mean of 0.31 ± 0.75 (mm)	G. APEX: mean of 0.08 ± 0.26 (mm)	G. APEX: mean of 1.68 mm ± 0.59		N. S	
Mittmann et al., 2020 [51]	13/16 (81,3%)	7/16 (44%)	9 cases	0.96% ± 9.61%	6.91% ± 12.36%	36.94 % ±57.42%	12/13	10/13	REAB 9/16 (56%), Ankylosis 5/16 (31%), Alveolar bone loss 2/16 (13%).
Nagata et al., 2014 [44]	23/23 (100%)	22/23 (95%)	1 case in G.CHP	N. S	N. S	N, S	G.TAP 10/12 (83.3%)<br>G.CHP 3/11 (27.3%)	0/23 (0%)	-----------
Nazzal et al., 2020 [41]	12/12 (100%)	10/12 (83%)	2 cases	-0.26 mm ± 4.54 (-1.7% ±30)	0.5 mm ± 0.91 (22.4% ±40.8)	1.33 mm ± 1.1	5/12 (33%)	5/12 (33%)	Slight tenderness to percussion 2/12 (17%)
Pereira et al., 2020 [49]	G. APEX 21/22 (95.46%)	G. APEX: 21/22 (95.46%)	1 case in G.APEX	G. APEX: 0.88 mm ±0.71 (6.6%)	G. APEX: 0.03 mm ±0.07 (0.99%)	G. APEX: 0.6 mm ±0.51 (30.47%)	G. APEX 0%	0/22 (0%)	G. APEX REAB, 2/22 (9,1%)
	G.RET 21/22 (95.46%)	G.RET: 21/22 (95.46%)	1 case in G.RET	G.RET: 1.42 mm ±1.25 (12.55%)	G.RET: 0.21 mm ±0.35 (6.7%)	G.RET: 0.88 mm ±0.77 (40.51%)	G.RET 9/22 (40.9%)		G. RET REAB, 7/22 (31.8%)
Rizk et al, 2019 [46]	25/25 (100%)	25/25 (100%)	------	PRP: 1.48 ± 0.37 mm (9.88% ± 2.85%)	PRP: 0.97 ± 0.75 mm (39.27% ± 32.04%)	PRP: 2.49 ± 3.93 mm (64.83% ± 18.5%)	N. S	0/25 (0%)	---------
				PRF: 1.24 ± 0.54 mm (8.19% ± 3.64%)	PRF: 1.003 ± 0.392 mm (42.37% ± 16.49%)	PRF: 1.73 ± 0.665 mm (76.75% ± 8.5%)			
Saoud et al., 2014 [45]	20/20 (100%)	20/20 (100%)	---	5%	21%	79%	N. S	0/20 (0%)	-----
Wikström et al., 2022 [52]	52/56 (92.9%)	45/56 (80.4%)	11 cases	1.17 mm (CI 0.93; 1.51), 11%	0.83 mm (CI 0.46; 0.88), 30%	Closed apex: 33 (73.3%),<br>Partially closed apex: 9 (20%).	8/45 (17.8%)	N.S	Ankylosis 2/56 (3.5%), periodontal pocket & mobility 1/56 (1.78%), persisting apical periodontitis 7/56 (12.5%)
Ulusoy et al, 2019 [53]	71/73 (97.2%)	71/73 (97.2%)	2 cases (1 PRF group, 1 BC group)	PRP: 5.11% ± 1.30 (%)<br>PRF: 7.05% ± 1.39 (%)<br>PP: 5.30% ± 1.41<br>BC: 5.06% ± 1.47	PRP: 19.48% ± 3.62<br>PRF: 11.14% ± 3.89<br>PP: 9.99% ± 3.92<br>BC: 12.25% ± 4.09	Complete apical closure: 73.9% (n = 54),<br>Continuous apical closure: 4,1% (n = 3)	N. S	63/73	----------
Zeng et al, 2022 [47]	34/34 (100%)	33/34 (97%)	1 case	Observed in 16 out of 34 cases with trauma	N. S	Observed in 22 out of 34 cases with trauma	N. S	N. S	------

G. APEX apexification group, G.RET revascularization group, PRP platelet-rich plasma, PRF platelet-rich fibrin, PP platelet pellet, BC induced blood clot, REAB root resorption, N.S not indicated, G.CHP calcium hydroxide and 2% chlorhexidine gel group, G.TAP triple antibiotic paste group.

Table S3. Results of the Egger test of each RET meta-analysis performed.

Studied Variable	Interceptor (CI 95%)	P value
Survival Rate	1.83 (0.89, 2.76)	0,001*
Success Rate	2.47 (0.62, 4.32)	0,01*
Signs of Apical Closure Event	2.17 (-3.18, 7.5)	0.3
Regain of Vitality	-4.03 (-7.8, -0.2)	0.04*
Crown Discoloration	0.79 (-4.6; 6.2)	0.72
Decrease in Apical diameter	2,68 (-8,14, 13,51)	0.48
Increase In Root Length	-1,04 (-2,54, 0,47)	0,13
Increase In Root Width	3,78 (-2,7, 10,3)	0,18

*: Significant.
Egger’s test indicates the existence of possible publication bias when the p-value is less than 0.05 (indicating significant asymmetry).
